# HIV-1 transmission: modelling and direct visualization in the third dimension

**DOI:** 10.1093/jmicro/dfad014

**Published:** 2023-02-10

**Authors:** Charles A Coomer, Sergi Padilla-Parra

**Affiliations:** Division of Structural Biology, Wellcome Centre for Human Genetics, University of Oxford, The Henry Wellcome Building, Roosevelt Drive, Headington, Oxford OX3 7BN, UK; University of Kentucky College of Medicine, Lexington, KY 40506, USA; Division of Structural Biology, Wellcome Centre for Human Genetics, University of Oxford, The Henry Wellcome Building, Roosevelt Drive, Headington, Oxford OX3 7BN, UK; Department of Infectious Diseases, Faculty of Life Sciences & Medicine, King’s College London, Floor 2, Borough Wing Guy’s Hospital, London SE1 9RT, UK; Randall Division of Cell and Molecular Biophysics, King’s College London, Hodgkin Building, Guy’s Campus, London SE1 1UL, UK

**Keywords:** HIV-1 entry, imaging HIV-1 in tissue, two-photon microscopy, two-photon FLIM metabolism, mucosal immunity

## Abstract

Identifying initial events of mucosal entry of human immunodeficiency virus type-1 (HIV-1) in laboratory-based, physiologically relevant and high-throughput contexts may aid in designing effective strategies to block local transmission and spread of HIV-1. Several paradigms have been posited for how HIV-1 crosses mucosal barriers to establish infection based on two dimensional (2D) culture–based or animal-based models. Nevertheless, despite these models stemming from 2D culture and animal studies, monolayers of cells poorly replicate the complex niche that influences viral entry at mucosal surfaces, whereas animal models often inadequately reproduce human disease pathophysiology and are prohibitively expensive. Organoids, having never been directly utilized in HIV-1 transmission investigations, may offer a compromise between 2D culture and animal models as they provide a platform that mimics the biophysical and biochemical niche of mucosal tissues. Importantly, observation of events downstream of viral inoculation is potentially accessible to researchers via an array of microscopy techniques. Because of the potential insights organoids may provide in this context, we offer this review to highlight key physiological factors of HIV-1 transmission at common mucosal sites and a discussion to highlight how many of these factors can be recapitulated in organoids, their current limitations and what questions can initially be addressed, particularly using a selective inclusion of quantitative light microscopy techniques. Harnessing organoids for direct observation of HIV-1 entry at mucosal sites may uncover potential therapeutic targets which prevent the establishment of HIV-1 infection.

## Introduction

Human immunodeficiency virus type-1 (HIV-1) replicates most efficiently in activated CD4^+^ T cells, the depletion of which leads to patients succumbing to opportunistic infections which characterize acquired immunodeficiency syndrome. Since its identification, HIV-1’s worldwide burden has exponentially expanded. In 2021, global projections estimated ∼38.4 million patients to be infected with HIV-1 [1], contributing to ∼650 000 deaths [1], and depleting up to 20% of health-care resources in significantly affected countries [2]. To date, there is no cure or vaccine for HIV-1 infection. The only available treatment is combination antiretroviral therapy, which halts progression to acquired immunodeficiency syndrome and allows infected patients to live to near-normal lifespans. Male circumcision campaigns and pre-exposure and post-exposure prophylaxis implementation have demonstrated encouraging results in preventing HIV-1 infection in clinical trials [3]. Furthermore, a plethora of immunology and vaccine studies have identified correlates of protection from infection at mucosal sites, which may catalyse the production of more efficacious prevention strategies [4]. In this context, it is critical to investigate the key underpinning mechanisms involved in HIV-1 penetration of the mucosal barrier to design novel therapeutic strategies that can efficiently prevent HIV-1 infection.

A variety of models have been posited as to how HIV-1 bypasses mucosal barriers and subsequently initiates acute infection, particularly at the cervicovaginal and colorectal mucosal surfaces [5]. For example, it is accepted that one method of transmission in the female reproductive tract involves HIV-1 infiltrating up to 10 µm of the squamous epithelium [6], where activated CD4^+^ T cells reside [7,8]. Contrastingly, HIV-1 can usurp a network of dendritic cells (DCs) in the distal rectum, triggering these DCs to migrate between rectal epithelial cells, capture the virus and transfer it to target activated CD4^+^CCR5^+^ T cells in the lamina propria [9–12]. Nevertheless, despite the high volume of literature investigating the mucosal entry of HIV-1, the real-time, direct infection of HIV-1 in target cells at mucosal surfaces and subsequent consequences has not been observed, owing to the heavy reliance of the current literature on animal and 2D culture models or *in vitro* techniques.

## Animal modelling of HIV-1 transmission: successes and pitfalls

Although animal models, particularly non-human primates (NHPs), have enabled historic progress in our understanding of mucosal penetration of HIV-1 and the development of effective prophylactic compounds (reviewed in ref [13]), NHP models harbour enormous variability regarding disease outcomes. This variability is dependent on the macaque species, chosen virus strains and the genetic pedigree of the individual monkeys [13]. Furthermore, NHP models are often prohibitively expensive, possess higher viral loads and require high inoculation titres [12].

Humanized murine models have also been paramount for understanding HIV-1 transmission due to efficient reconstitution of mouse rectal and vaginal mucosa with human immune cells [14]. Additionally, humanized mice have allowed researchers to compare various antiretroviral therapeutics to prevent transmission *in vivo* [14], such as the nucleoside reverse transcriptase inhibitor EFdA [15] and long-acting antiretroviral drugs to facilitate pre-exposure prophylaxis adherence. In particular, one study using a single injection of the long-acting integrase inhibitor raltegravir in NSG-BLT mice demonstrated protection to HIV-1 challenges up to 4 weeks after administration [16]. Various methods for antibody administration to prevent HIV-1 transmission (e.g. topical application [17], adeno-associated virus vector immunoprophylaxis [18] and passive immunization [19,20] have been tested in humanized murine models as well.

Nevertheless, despite these advances, humanized murine models are met with limitations. In particular, these models often suffer from impaired secondary lymphoid tissue development, suboptimal engraftment, species-specific cytokines and the need for human leucocyte antigens for appropriate T cell selection following haematopoietic stem cell engraftment (reviewed in ref [23]). Additionally, graft-versus-host disease (GVHD) often develops ∼6 months after engraftment, and this phenomenon limits the use of the humanized mouse in studies analysing the efficacy of long-term immunization regimens. Furthermore, early development of GVHD may alter T and B cell activation states, which may impact HIV-specific cellular and humoral responses at mucosal sites during transmission [21]. Interestingly, blocking HIV-1 transmission in humanized murine models has been deemed more achievable relative to humans because of qualitative and quantitative differences of the human immune cells present in the mouse mucosa compared to normal human mucosa [14]. However, other investigators have shown that human cells in the murine female genital tract (FGT) are subjected to an oestrous cycle that could result in increased numbers of activated CD4+ cells than would be found in human females [22], which would be thought to increase the frequency of HIV-1 transmission. Ultimately, either animal model precludes the direct and real-time observation of HIV-1 transmission, which most likely would require a sufficiently physiologically relevant, high-throughput tissue model that could be multiplexed with label-free microscopy techniques, which could indeed revolutionize HIV-1 transmission studies.

## Meeting in the middle: 3D tissue models to study initial steps of HIV-1 transmission

Although much has been learned from 2D culture models, these systems suffer from similar constraining inadequacies. For example, monolayer systems lack tissue architecture and the mucosal-specific biochemical niche (e.g. cytokines, microbiota and host secretions) that could influence the success of viral infection establishment [23]. Furthermore, these anatomical and immunological factors likely alter the number of available target cells, their activation status and accessibility and dissemination, which could equally affect their likelihood of infection.

**Fig. 1.**
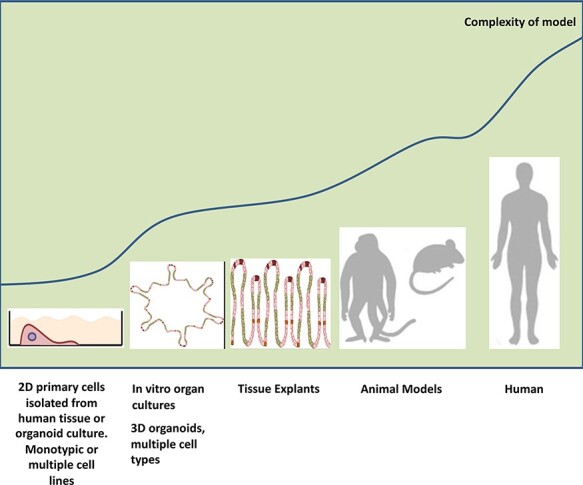
Current biological experimental systems and their relative level of increasing complexity in reproducing human infectious pathology. Schematic representation of the current culturing techniques available and their ability to reproduce the complexity of human–HIV interactions: from 2D monolayer cancer cell lines to human studies. Human cancer cell lines have played a vital fundamental role in determining underpinning pathways involved in HIV transmission and have been critical in screening and characterizing therapeutic candidates to prevent or treat acute HIV infection. Additionally, these cells have been utilized to investigate how HIV-1 may interact with epithelial cells at the mucosal barrier. Similarly, primary cells isolated from human tissue or organoid cultures have also been paramount in generating more physiologically relevant data to answer similar questions. Nevertheless, these two models fail to recapitulate the *in vivo* 3D microenvironment, induce cell differentiation, possess natural tissue-specific cell heterogeneity and perform tissue-specific functions. Oftentimes, many of these limitations are bypassed using other models such as fixed tissue explants or animal models in order to get closer to mimicking human HIV-1 pathophysiology. Nevertheless, fixed tissue models often only offer indirect evidence to support certain investigations and the fixing and explanting process may alter variables (e.g. cell migration) critical for the particular scientific question. Investigations using NHP models are dependent on the species, viral strains used for infection, genetic pedigree of the individual monkeys and humanized murine models are met with challenges regarding poor lymph node development, humoral responses, engraftment and species-specific cytokines. To balance this spectrum of experimental platforms, their benefits and drawbacks, organoids enable the mimicry of the intricate 3D organization and architecture of tissue, tissue-specific functions, natural host microbiota and the cell heterogeneity found in natural tissue in addition to being reproducible and subject to live-cell imaging. Therefore, organoids offer a compromise on the tissue culture platform spectrum between 2D monotypic cancer cell lines and living organisms.

**Fig. 2.**
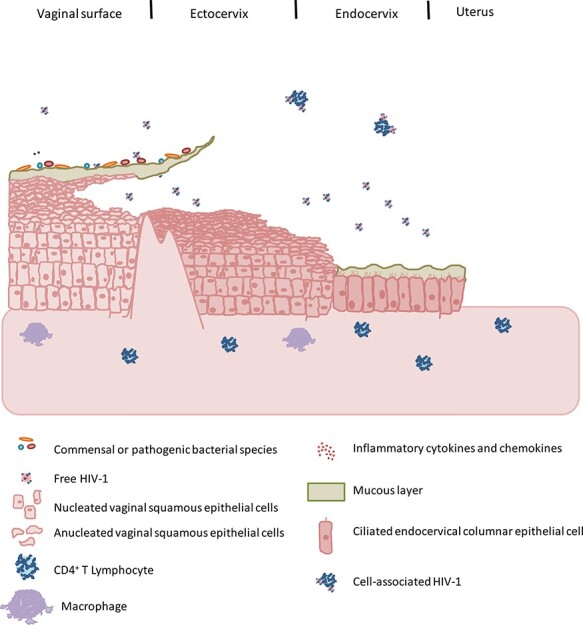
Key routes of HIV-1 penetration in the FGT. The human vagina and ectocervix are insulated by a multi-layered stratified, non-keratinized squamous epithelium. Nevertheless, HIV-1 can penetrate the FGT via multitudinous mechanisms, the following of which that are mentioned form a non-exhaustive list. (a) Free or cell-associated HIV virions may become trapped in cervicovaginal mucous. These mucous-trapped cells and virions may then subsequently have sufficient exposure time to the epithelial surface, such that free virions or virions produced by attached HIV-infected donor cells may navigate through gaps between epithelial cells, be captured and transcytosed by epithelial cells or be captured and trafficked to endocytic compartments by epithelial-resident Langerhans cells. (b) Free virions or virions from infected donor cells may fuse with intraepithelial CD4+ T cells. (c) Cell-associated virions may be transmitted to epithelial cells which may then be transcytosed to later target and infect underlying stromal macrophages. Cell-associated virions may also be transmitted to CD4+ T cells via the virological synapse. (d) During sexual intercourse, physical abrasions of the epithelium commonly occur, often in regions where stromal papillae projections, which are enriched in immune cells, nearly reach the luminal surface. Importantly, potentially free virions or cell-associated virions from infected donor cells may pass through these abrasions to directly contact the plethora of target cells within the mucosa or stroma. Because resident HIV-1 target cells cluster in these regions, numerous infection foci are created. (e) Stromal projections also are docking points for lymphatic and arteriovenous vasculature and can therefore transport cell-free or cell-associated virions to the systemic circulation or local lymph nodes. (f) Conflicting reports indicate that it is possible that free HIV-1 virions may transcytose through or productively basal epithelial cells throughout the FGT, including uterine epithelial cells (not pictured). (g) Viruses within the stroma are capable of being taken up by or (conflictingly) productively infect stromal DCs in order to subsequently transfer virions to susceptible CD4+ T cells via the viral synapse, followed by an explosive increase in productive infection, whereby generated virions can continue to infect other susceptible cells (e.g. macrophages). (h) Cytokines produced by stromal leucocytes and epithelial cells in response to commensal bacteria or sexually transmitted infections may act to recruit HIV-1 susceptible target cells to increase the target population of susceptible leucocytes. (i) The junction between the stratified squamous epithelial barrier that characterizes the ectocervix and the high-turnover, single-layer columnar epithelial layer of the endocervix, is known as the transformation zone. Because of its delicate monolayer, the endocervix may be the preferential site of HIV-1 entry in the FGT.

DCs, dendritic cells; FGT, female genital tract; HIV, human immunodeficiency virus; HIV-1, human immunodeficiency virus type-1.

The utilization of organoid-based models can help bridge the gap between 2D cell culture and animal model systems to study viral entry at mucosal surfaces (Figs. 1 and 2). Named the method of the year 2017 by *Nature Methods* [24], organoids are stem-cell-derived (pluripotent- or adult-derived), self-organizing, three-dimensional (3D) multicellular *in vitro* platforms that closely mimic their *in vivo* tissue counterparts. First having been used to generate components of the central nervous systems [25], organoids can now be used to construct a plethora of different tissues. Because they can be expanded while maintaining their tissue identity and architecture, these systems can be harnessed to model a variety of human pathological states. In the context of viral infection, organoids allow the added complexity of multiple cell–cell interactions. This has been demonstrated in hepatitis C infection, where it was shown that the interactions of supporting cell types (e.g. Kupffer cells and endothelium) with host hepatocytes influenced the course of their infection [26,27]. Organoids also allow the construction of spatially defined tissue environments encompassing multiple tissue compartments, such as the skin and nervous tissue in herpes simplex virus type-1 infection, which can incorporate time-lapse imaging [28].

3D, three-dimensional; HIV, human immunodeficiency virus; HIV-1, human immunodeficiency virus type-1; NHP, non-human primate.

In a field in which human explant and co-cultivation cultures are pervasive when studying HIV-1 transmission, organoids could be utilized to further the insights obtained by these investigations. For example, explant models and co-cultivation studies have demonstrated the capability of HIV-1 to bind and enter epithelial cells of the lower FGT and be transferred to susceptible CD4^+^ T cells [29–32]. However, in tissue explants, infection of subepithelial mucosal cells is often conducted via explant inoculation immersion. Therefore, infection is no longer exclusive to the epithelial surface in traditional infection. Other infection models of polarized tissue have been performed such as culture air–liquid interface [33], embedding in agarose [34] or placing tissue on sponges [35], although the latter is typically achieved similarly by submerging tissue in cell-free virus, thus not faithfully modelling natural infection. Additionally, many cell types, and in particular DCs and Langerhans cells, often migrate outside of and away from the tissue explant, making it impossible to distinguish between the migrant cells of intraepithelial origin and the immune cells of the stroma in HIV-1 transmission studies when trying to determine initial cells infected during HIV-1 transmission [36,37]. Moreover, rapid loss of the structure and integrity of stratified organotypic explants is often observed [37]. Thus, prompt handling after surgeries to transiently retain physiologic relevance of tissue architecture and cellular interactions is required and is prohibitive to long time-course experiments [38]. Furthermore, these systems are inherently not self-renewing and require patient donors. Thus, these model systems, though useful, highlight a meaningful gap in studying virus–host interactions. Consequently, a clear-cut method of visualization of transmission utilizing advanced imaging and a high-throughput 3D model of mucosal surfaces is needed.

Organoids are beginning to bridge this gap as a self-renewing, high-throughput culturable alternative to explanted systems. As self-organized, 3D structures consist of multipotent tissue-specific stem cells and their differentiated progeny, these *in vitro* systems can be propagated virtually indefinitely provided viable culture conditions [39]. Intestinal and colonic organoids recapitulate *in vivo* tissue architecture, with crypt-villous domains with the full spectrum of differentiated epithelial cells [40] and mesenchymal cells in pluripotent stem cell–based platforms [41]. Interestingly, although several RNA-sequencing investigations have shown that intestinal and colonic organoids faithfully recapitulate native intrinsic transcriptional programmes [42–44], *in vitro* culture strongly influences the global transcriptome, and several transcriptome features may be lost after culturing [42,44]. Newer to the field, organoid systems of the FGT are emerging, particularly of the cervix [45,46], showing reliable recapitulation of gene expression profiles of native tissue [45] and the ability to be maintained in culture for several months with generation of the transition zone [45,46].

**Fig. 3.**
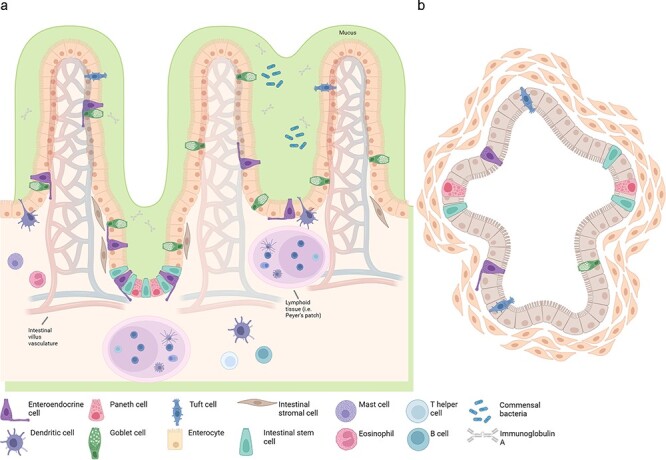
Comparison of intestinal tissue explants vs. their organoid counterparts. (a) Structure and cell type specification of explanted intestinal epithelium. The intestinal epithelium consists of stem cells, Paneth cells (small intestine), goblet cells, tuft cells, enteroendocrine cells, enterocytes (small intestine)/colonocytes (colon), stromal cells and microfold cells (not pictured) overlying lymphoid tissue. Importantly, explanted tissue often temporarily preserves lymphatic tissue and microvasculature (pictured), however immune cells often emigrate out of the explants early. (b) Pluripotent stem-cell-derived intestinal organoids, although containing intestinal epithelium consists of stem cells and their derivatives, in addition to mesenchymal cells, mucous and any co-cultured cells (which varies and is thus, not pictured). Created in *BioRender*.

However, there are several structural limitations of these platforms to keep in mind. While intestinal and colonic organoids harbour epithelial and mesenchymal cells, they form a luminal structure which lacks complex mesenchymal heterogeneity and architecture compared to native and explanted tissues, such as vasculature, neuronal connections, immune milieu and microbial flora. Although co-culture strategies with these components exist to increase organoid complexity, these systems are still relatively simple [39,47–49], combining merely one or a few cell types. Furthermore, many conventional organoid cultures lack the presence of *in vivo* growth factors, complex extracellular matrix composition and fully optimized biomechanical properties of their environment (e.g. peristalsis) [50]. Additionally, inherent to all organoid models is their intrinsically high degree of variability, as tissue source heterogeneity, tailored media components, inconsistent passaging methods and somewhat random positioning in matrices construct a stochastic system for experimentation [51]. Thus, careful consideration of organoid systems must be balanced when comparing them to native tissue explants (Fig. 3).

Arguably, the most attractive aspect of organoids is their synergy with advanced imaging approaches, owing to the fact that multiple imaging platforms were exploited to determine that organoid complexity and morphology faithfully mimics *in vivo* equivalents. In particular, non-invasive optical sectioning methods such as two-photon confocal laser microscopy enable the visualization of fine cellular detail and tissue architecture [52]. Additionally, advanced confocal and light sheet microscopy may also be utilized to probe global organoid structure, cellular positioning and single-particle tracking, the latter particularly useful for minimizing light exposure and phototoxicity when tracking and quantifying virus transmission events [52].

Organoids combined with advanced imaging could be ideal models to study mucosal invasion. A key unanswered question in HIV-1 pathogenesis remains how the virus is able to overcome the epithelial barriers of the genital and rectal mucosae during sexual transmission. Furthermore, the defined numbers and characteristics of the various mucosal cellular targets necessary for HIV-1 to initiate a productive infection remain incompletely characterized. Microscopic imaging in these complex, organotypic models will enable such investigations, as well as address additional questions, such as whether luminal entry into the epithelium through diffusive percolation is sufficient for HIV-1 to reach its early target cells, or if donor or host-derived HIV-1-carrying or -infected cells migrating transepithelially prevail.

## Organoids of the FGT and colorectal tract provide a high-throughput, physiologically relevant model to investigate HIV-1 entry

As mentioned earlier, organoids are stem-cell-derived organized 3D structures that attempt to mimic both the structure and the function of organs. These stem cells are typically derived from biopsies isolated directly from the patient’s tissue of interest (i.e. adult stem-cell-derived organoids) or generated from induced pluripotent stem cells, the latter of which allowing the banking of patient-derived stem cells [53,54]. Importantly, organoids have become an important tool to study host–pathogens interactions [55], including human viruses [56]. There are two types of intestinal organoids that have been used for virus studies: one that derives from resident proliferating stem cells (PSCs) isolated from biopsies or surgical tissues and the other that derives from pluripotent stem cells. As expected, both types of organoids consist of intestinal epithelium, but they differ in the presence of associated stromal mesenchyme that only is expressed in organoids derived from PSCs [56–58].

Because of the excess of conflicting reports regarding initial HIV-1 target cell entry and the subsequent events that follow their infection, a high-throughput, physiologically relevant method enabling the direct observation of infection of cells and the consequential events in the FGT and colorectal tract (CRT) is vital. Several models which investigate the influence of the anatomical structure, extracellular milieu or neighbouring cells on the success of HIV-1 infection of target cells have offered evidence that both complements and opposes previous cell culture–, tissue explant– and animal model–based studies, and this may be because mimicking these physiological conditions is required to accurately represent the susceptibility of key target cells to HIV-1. If so, these studies reinforce the notion that organoids may pave the way for elucidating the initial events of HIV-1 infection in the FGT and CRT as they are capable of mimicking native architecture, target cell distribution and immunological milieu of human tissue. Moreover, identifying these seminal events of HIV-1 infection at these mucosal sites may uncover key targets for curtailing HIV-1 transmission at these sites or treating acute HIV-1 infection.

For example, when considering the influence of the laboratory-based milieu on HIV-1 target cells in 2D culture, the most fundamental components such as O_2_ tension can alter the frequency of HIV-1 infection. Specifically, O_2_ tension in lymphoid organs and stromal tissue can be as low as 0.5–4.5%, in contrast to 21% in 2D culture incubation, which drastically increases the levels of Glutamate 1 on the cell surface, enriching HIV-1 susceptibility of quiescent CD4^+^ T cells, which have been previously shown to be non-susceptible [59]. Organoids of the FGT or the CRT may model O_2_ tension more closely to *in vivo* as O_2_ becomes less likely to penetrate deeper layers of these 3D constructs [60], generating physiologic hypoxia that exists in deeper layers of the stratified squamous epithelium of the FGT or the colon, as well as the lamina propria of the endocervical epithelium or rectal canal. In fact, studies have already shown that in 200-µm spheroidal organoids, O_2_ tension borders physiologically hypoxic zones ∼100 µm from the centre of the tissue [61].

In addition to modelling O_2_ tension, functional organoids can produce mucous, which can trap seminal cells or free virions, which may facilitate virus transmission via the accumulation of contact time between free virions or infected cells with the mucosal surface [62]. This could be of particular relevance to HIV-1 infection in both the FGT and CRT which have substantial mucous production. Furthermore, organoids can further mimic *in vivo* conditions by artificially supplementing physiologically relevant levels of hormones, such as progesterone and oestrogen into the culture medium, where high levels of progesterone in the secretory phase of the menstrual cycle has been shown to create increased vulnerability to HIV-1 infection by vaginal epithelium thinning and suppressed mucosal immunity [63,64]. Moreover, organoids offer an opportunity to elucidate the influence of neighbouring cells on the success of HIV-1 infection in the FGT and CRT, a topic in which the current literature is debated. For example, intestinal organoids can differentiate into multiple cell lineages, such as enteroendocrine cells, M cells, Paneth cells and mucous-secreting mature goblet cells [65], which could not previously be maintained in culture [66]. Thus, key questions regarding HIV-1 interactions with these neighbouring cell types and their influence on the mucosal milieu can be addressed.

The added complexity of the endogenous inflammatory milieu, commensal microbiota and target cell densities are all additional parameters which can be investigated in a more-controlled environment with an organoid-based platform. Indeed, there is a considerable gap in the literature investigating HIV-1 mucosal transmission in the context of the intricate cytokine and chemokine milieu at transmission sites. Additionally, although studies investigating the role of cell intrinsic immunity (e.g. pattern recognition receptors) in HIV-1 infection in tissue culture systems have provided considerable insight into HIV-1 protection and pathogenesis, expansion of these studies to organoid-based platforms is lacking. This presents a considerable opportunity to evaluate the role of cellular innate immunity in 3D systems in a high-throughput setting, as not only do intestinal, colonic and cervical organoids express pattern recognition receptors at relatively similar levels compared to native tissue [67–69] but also respond similarly when triggered by cognate pathogen-associated molecular patterns, thus providing opportunities to mimic the inflammatory milieu in native tissue [67–69]. In organoid systems which incorporate commensal or pathogenic microbiota, it is conceivable that investigating the role of their detection and inflammatory consequences in HIV transmission in a high-throughput 3D system can be envisaged. Furthermore, understanding the cellular innate immune system and the corresponding viral evasion mechanisms during infection in HIV-1 transmission in organoids may provide important guidance for the design of adjuvant and vaccine strategies [70,71]. Importantly, these investigations will be further enhanced when future organoid technology is able to routinely and faithfully recapitulate all immune cell populations in native tissue.

Much sparser is data investigating the role of commensal organisms that reside in either the FGT or CRT on the course of HIV-1 transmission. Thus, investigations analysing the effects of each of these parameters in organoid models are warranted. By extension, it is likely that organoids can expand our understanding as to how comorbid infections within the FGT or CRT influence HIV-1 transmission in these tissue compartments. Currently, literature which has studied such infections in organoids is lacking (i.e. hepatitis C, human cytomegalovirus, tuberculosis, candida and gonorrhoea). Nevertheless, we believe that establishing organoid models for these infections will pave the way to study comorbid infections in HIV-1-infected tissues.

It is important to mention however that organoids are not without their shortcomings. One of the most obvious drawbacks of PSC-derived intestinal organoids is that they do not yet innately support the generation of immune cell populations, which is prohibitive for investigations modelling HIV-1 infection. Nevertheless, developed co-culture systems faithfully allow several different immune cells to be incorporated separately, including antigen-presenting cells (APCs) such as DCs [72,73] and macrophages [74,75], as well as lamina propria–derived T cells and their subsets [76–78]. As organoids do not have stromal tissues or lamina propria, often these immune cells cultured with organoids are supported in a gelatinous basement membrane mixture derived from sarcoma cells (i.e. Matrigel) [65,79], which is not ideal for leucocyte motility, chemotaxis and facilitating durable immune–epithelial cell interactions [79]. However, synthetic hydrogels have now paved the way to improve immune cell–epithelial interactions and organoid durability [79–81]. Furthermore, several groups have now generated mechanically disrupted organoids which preserve crypt-physiology and a polarized intestinal lining, generating a lumen apical domain and counterposing basal lamina propria [82,83]. Encouragingly, this model has been shown to be effective in evaluating pathogen–epithelial–immune cell interactions, even capturing APC-derived dendrites extending through the intestinal epithelium to interact with the pathogen of interest [82,83]. Although co-culturing multiple immune cell populations (e.g. APCs and T cells) with organoids has been previously durably achieved [84,85], thus allowing a focus for HIV-1 infection, this has not, to our knowledge, been conducted within the scope of host–pathogen interactions.

Collectively, as organoids continue to better recapitulate the immune microenvironment, it will be possible to utilize these co-culture platforms to analyse the initial steps of HIV-1 infection in the intestinal tract and even generate a focus of infected target cells. Indeed, HIV-1 is thought to penetrate the colorectal tissue and generate a focus of infection via a plethora of ways, including (I) transiently open tight junctions of intestinal epithelial cells, (II) transcytosis across the epithelial cells, (III) binding dendritic APC extensions between epithelial cells and (IV) capture by cells in the lamina propria via microabrasions in the epithelial lining. Lamina propria CD4+ cells are thus directly infected or more likely captured by APCs and transmitted to neighbouring CD4+ T cells [11]. By combining APCs and target CD4+ T cells in polarized 2D and 3D organoid models, the direct visualization of these initial steps can potentially be easily visualized in real time in a high-throughput model which could subsequently evaluate effectiveness of therapeutics which deter transmission (e.g. microbiocidals). Nevertheless, depending on the platform utilized, not all modes of HIV-1 transmission may be visualized or foci of infection generated. Thus, careful consideration of the experimental questions to be asked and model selection is required.

Another pitfall is that despite a variety of organoid systems becoming increasingly available, many systems utilize unique culture conditions. These conditions may or may not impact experimental outcomes depending on the investigation in question. What is more, there is a distinct lack of reports which scrutinize and compare organoid culture systems to determine which model is ideally suited for certain scientific investigations. Although several RNA-sequencing studies of organoid systems have been conducted [86–88], those that analyse the influence of heterogenous culturing conditions and how they influence gene expression profiles, cell populations, cell intrinsic immune status and immune milieu are required. These studies will be capable of illustrating how well represented a particular tissue segment is, which cell types are present, how well differentiated they have become and how long they maintain these properties relative to native conditions.

Until recently, organoid models were generated while devoid of supportive cells normally present in native microenvironments, such as mesenchymal and stromal cells, immune cells and blood vessels (Table 1). Indeed, CRT organoids once generated from biopsies or surgical tissues merely contained mostly epithelial cells [56]. In the context of HIV transmission, organoids devoid of specific target cells for the virus, such as CD4+ T cells, would obviously diminish the impact of the findings. In the case of HIV-1 infection, development of co-culture conditions of organoids and immune cells foreshadowed the next frontier of organoid biology. Indeed, organoid systems utilized in the colonic, cervical and several other cancer studies have now incorporated immune cells, including PMBCs and stromal cells. Although in its infancy, organoids incorporating immune cells have already been utilized to study HIV-1 infection and pathogenesis.

**Table1.** Comparison of several key advantages and disadvantages of the major animal and tissue models for HIV-1 transmission research

	2D immortalized culture models	Primary cultures of cells	Co-culture	3D reconstructed stratified mucosa	Tissue explants	Humanized mice/HIV	NHP/SIV or SHIV	Organoids
Sample size	++++	++++	++++	++++	++	+	+	++++
Costs	+	+	+	++	++	+++	++++	++
Accessibility	++++	+++	+++	++	++	+	+	+
Similarity of virus to HIV used	Similar	Similar	Similar	Similar	Similar	Similar	Different	Similar
Procedure required to generate model	No	Phlebotomy	Phlebotomy (potentially)	Phlebotomy (potentially)	Surgical biopsy	Surgery	No	Surgical biopsy if tissue-derived
Development of GVHD	N/A	N/A	N/A	N/A	N/A	Yes	No	N/A
Reproducibility	Well characterized and standardized	Well characterized and standardized	Well characterized and standardized	Well characterized and standardized	Well- characterized and standardized	Well characterized and standardized	Well-characterized and standardized	Not as well characterized or standardized
Time to produce	Minimal, fully polarized and differentiated cells take 8–10 days	Minimal, fully polarized and differentiated cells take 8–10 days	Minimal, fully polarized and differentiated cells take 8–10 days	5–8 weeks to obtain reconstruction	Immediately after surgery and sectioning is performed	Months to establish a colony, 12–14 weeks to verify engraftment	May begin transmission studies upon acquisition	Tissue-derived organoids may take less than 1 week, iPSC/ESC-derived may take >1 month
Problems with viability	Indefinitely	2–4 weeks	Depends on the cells incorporated	Depends on the cells incorporated	Tissue architecture can quickly collapse, immune cells can rapidly emigrate	GVHD from immune cell reconstitution	Not limited in studies of HIV-1 transmission	May self-renew, so can be expanded for months, some models indefinitely
References for review	Ref [5,27,41]	Ref [5,27,41]	Ref [5,27,41]	Ref [5,27,41]	Ref [5,27,41]	Ref [13,17,23,25,26]	Ref [13, 14, 25]	Ref [27,41,96,97]

GVHD, graft-verus-host disease; HIV, human immunodeficiency virus; HIV-1, human immunodeficiency virus type-1; N/A, not applicable; NHP, non-human primate.

Encouragingly, there continues to be progress in co-culture models incorporating other cell types, such as endothelial cells and immune cells, in intestinal organoids. An efficient *in vivo* engraftment model of human pluripotent stem cell-derived organoids was generated to facilitate the development of a functional vasculature system in cerebral organoids, and similar models could be envisaged for the development of vasculature systems in colon, intestinal and FGT organoids [89]. Additionally, Noel *et al*. have described primary human macrophage-enteroid and colonoid co-culture systems, which could serve as a preliminary platform to assess the transmission of R5-tropic HIV-1 to macrophages in the colon during CRT transmission of HIV-1 [83,90]. 3D co-culture systems of intestinal organoids and DCs, although initially designed to study epithelial differentiation [91], could also be utilized to study HIV-1 interactions with DCs during transmission in tissue via live-cell imaging, and perhaps extension of this co-culture method could be harnessed to analyse macrophages and DCs together during HIV-1 transmission in these previously published co-culture systems.

Encouragingly, Nozaki *et al*. have demonstrated the co-culturing of intestinal organoids with purified intraepithelial lymphocytes and studied their migration with fluorescence microscopy [92]. These studies are critical as the implications of γδ T cells (i.e. Vδ2 T cells) in HIV-1 infection are poorly defined, despite being reported as susceptible to HIV-1 [93,94] and simian immunodeficiency virus infection [95], and contribute to the latent HIV-1 reservoir [96]. We believe that the utilization of intestinal and colonic organoid models co-cultured with these intraepithelial lymphocytes will help pinpoint several questions regarding HIV-1 entry and their role in HIV-1 transmission and infection. What is more, organoid models addressing HIV-1 chronic infection also may theoretically be possible, as current organoids of the intestine and endometrium demonstrate robust replicative and long-term potential.

Furthermore, as organoid technology is constantly evolving, we believe that the incorporation of other HIV-1-susceptible cells (i.e. DCs and T cells) into organoids of the CRT, intestine and FGT will become integrated into standard platforms in the very near future. Encouragingly, in a study analysing adaptive immune regulation of mammary organogenesis, Plaks *et al*. have successfully generated a co-culture system that enables the analysis of lymph node–derived or spleen-derived Th1 and Th2 CD4+ T cells and CD11c+ APCs [97]. Additionally, Purwada *et al*. have been able to generate B cell follicle organoids that recapitulate lymphoid tissues capable of germinal centre reactions, B cell differentiation and induction [98,99], which could be utilized to assess questions regarding immunological dysregulation of lymph node during HIV-1, as well as to deduce the mechanism of follicles serving as sanctuary sites for HIV-1. Together, the utilization of currently modelled FGT and CRT organoids will facilitate studies on HIV transmission and new discoveries on virus–host interactions, including information about the mechanisms involved in chemokine-dependent recruitment of target cells, the role of tissue architecture in HIV-1 transmission, the effect of certain compounds and the role of the microbiota in the susceptibility to the infection. Furthermore, they could also be relevant models for preclinical drug evaluation, including vaginal microbicides to prevent transmission of HIV in women [100].

## Imaging techniques to visualize viral entry in organoids and tissue

In the last decade, light microscopy has gained momentum not only as a powerful technique to image cells in their natural environment (i.e. tissue) with improved spatial and temporal resolutions [101,102] but also as an advanced platform when combined with spectroscopy to quantitatively determine mechanistic aspects related to particular biological and pathological contexts, specifically infection [103,104]. As mentioned earlier, so far, the majority of studies dealing with HIV-1 infection in tissue have been performed employing confocal microscopy of fixed samples [34,105]. In this sub-section, we describe the light imaging techniques that we consider could have a decisive impact when assessing HIV-1 (and other viruses) infection in organoids and tissue in the next few years.

### Label-free two-photon confocal microscopy and intravital FRET-FLIM

**Fig. 4.**
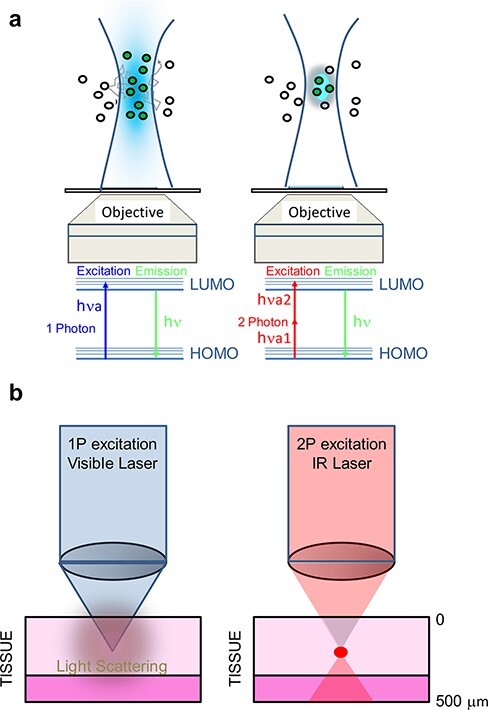
Principle of two-photon microscopy. (a) Two-photon microscopy (right panel) offers a number of advantages as compared with conventional sinlge-photon excitation approaches (left panel). In two-photon microscopy, the transition between the highest occupied molecular orbital toward the lowest unoccupied molecular orbital is excited by two photons simultaneously. The total energy of these two photons is the same as the one needed for one-photon excitation events (which depends on the particular fluorophore to be excited/studied). Longer wavelengths imply lower energy and therefore less phototoxicity. Also, less scatter is obtained with two- or three-photon microscopy enabling deeper tissue imaging (b).

**Fig. 5.**
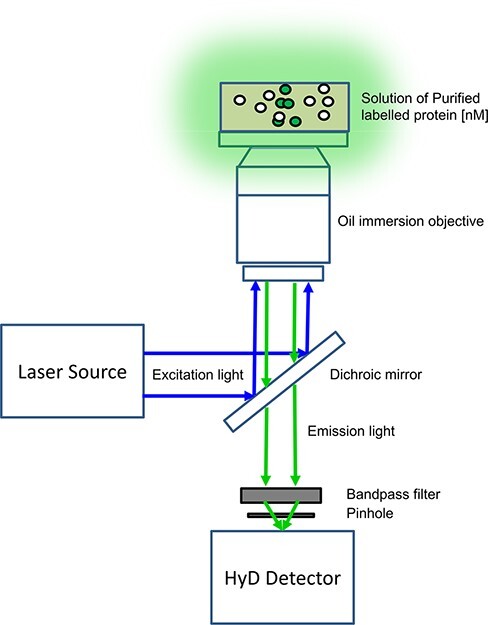
Principle of two-photon microscopy coupled to TCSPC FLIM. Coupling of two-photon excitation with TCSPC FLIM allows measurement of endogenous-free and -bound NAD(P)H relative concentrations in live cells and tissue. The optical path of a typical two-photon FLIM microscope is shown. A Ti:Sa pulsed femtosecond laser is directed toward the dichroic and is also coupled to a photo-diode and TCSPC electronics. The emitted photons arrive to the photon counting detector with a particular delay relative to the two-photon femtosecond pulsed (normally tuned at 80 MHz, i.e. one pulse every 12.5 ns). The computer provides the fluorescence decay or the right coordinates for the phasor plot (right panels). For non-fitting approaches, only around 100 photons per pixel are enough to recover the average lifetime and the minimal fraction of interacting donor if interested in detecting FRET between two fluorophores (donor and acceptor); for instance, FRET-based biosensors.

FLIM, fluorescence lifetime imaging; FRET, Forster resonance energy transfer; TCSPC, time correlated single-photon counting.

Multiphoton microscopy (Fig. 3) is a platform which can be utilized for label-free and non-invasive functional imaging in organoids and tissue [103]. In-depth imaging of tissue and organoids is normally achieved with pulsed, near-infrared excitation light taking advantage of (i) endogenous fluorescence [106,107], (ii) second harmonic generation [108,109] and (iii) third harmonic generation [110]. Endogenous fluorescence is related to the excitation of NAD(P)H and FAD molecules, both of which are very important cofactors of redox reactions and metabolism [111,112]. The combination of multiphoton microscopy with lifetime imaging (fluorescence lifetime imaging, FLIM) (Fig. 4) to specifically study NAD(P)H and FAD dynamic reports on the metabolic activity of individual cells and has been employed in different biological and pathological contexts, such as stem cell differentiation [113], cancer development [114] or neurodegenerative diseases [115]. The fluorescence lifetimes of NAD(P)H and FAD change according to their environment, that is, upon protein binding during the electron transport chain [116]. Label-free lifetime imaging of NAD(P)H and FAD therefore provides information on the ratio of free and bound NAD(P)H and redox states NADH/NAD^+^ of single cells within the tissue (Fig. 5). This information is in turn related to the glycolytic and the metabolic states of the cells.

Although this novel approach is attractive for general immunology investigations and dissecting host–pathogens (HIV-1) interactions, in particular, there remains an underwhelming absence in the literature of studies combining these measurements during infection. In the particular case of HIV-1, the importance of cellular metabolism and infection has been shown in multitudinous publications [117–120]. In particular, studies elucidating the role of glycolysis [121] and glucose uptake [118] in HIV-1 infection illustrate that the host capacity for increased glucose transport could condition CD4^+^ T cell and macrophage susceptibility to HIV-1 infection. The possibility of label-free imaging individual cell metabolism within infected tissue or even during infection would help to understand many questions related to innate HIV-1 immunity. These types of quantitative measurements are further warranted because CD4^+^ T cells sitting in observation chambers most likely do not behave the same as CD4^+^ T cells residing in a 3D matrix [122]. It is therefore very likely that their behaviour will be very different also in tissue and organoids.

### On-line single-particle tracking in 3D

An exciting and new implementation combining on-line 3D single-particle tracking [123] with two-photon microscopy was recently developed by Welsher *et al.* (2014): 3D multi-resolution microscopy. Thus far, the method has been applied for coated nanoparticles (100 nm) [124] in different cellular contexts (mainly endocytosis and transcytosis). However, given the fact that these particles are approximately of similar size to HIV-1 virions and the imaging platform is additionally equipped with a multiphoton laser, it should be—in principle—possible to image and track single HIV-1 particles in tissue. However, the probability to detect and track single HIV-1-labelled particles that would be subsequently infectious and productive in tissue, however, is very low [125] This implies that automation of these experiments (both acquisition and analysis) is required to provide important information about the fluorescence lifetime, the whereabouts and specific trajectories of infectious particles in physiologically relevant environments.

Encouragingly, Hou *et al*. have recently developed a real-time 3D single-particle tracking approach using a dynamic moving laser spot, called 3D-DyPLoT [124]. With this approach, the authors were able to track lentiviral particles decorated with VSV-G Env labelled with EYFP. The large observation area of this method (1 µm × 1 µm × 4 µm) and the improvement in detection sensitivity as compared to 3D multi-resolution microscopy make this technique a potential candidate to track fast single virions in tissue. It has to be said that scattering, bleaching and limited photon counts coming from single virions could challenge long-term observations and these prospects need to be validated in the laboratory.

### Light sheet microscopy

Light sheet fluorescence microscopy (LSFM) is an attractive approach for fast 3D sectioning of large tissue samples. Approximately a decade ago, Verveer *et al*. [126] showed that 140-µm spheroids of human pancreatic cells could be imaged with a non-invasive LSFM variant: selective plane illumination microscopy (SPIM). SPIM has since been recognized as a very powerful tool to image tissue samples since it provides fast 3D imaging with very low phototoxicity [127].

It is important, nonetheless, to stress a few drawbacks and limitations associated to the technique, such as optical aberrations, high absorption and scattering coming from the sample, the width and shape of the illumination sheet and side-illumination-related striped images. These limitations prevent SPIM to be a good candidate for deep tissue imaging. This is because typically, >100 µm, these artefacts are too important and have a relatively impactful distortion on the quality of the images acquired. There are, however, many research lines trying to alleviate these problems [128], and these investigations have uncovered many variants and approaches with their strengths and weaknesses. In the particular case of HIV-1 infection and quantitative imaging, we believe that SPIM might be too challenging at the present time to being able to decipher the particular point of infection or to follow single HIV-1 virions. Moreover, even if LSFM has been combined with quantitative imaging [129,130], it is still too early to consider deep imaging quantitative microscopy using this method, and therefore, further research is needed to make LSFM attractive to measure infection in live tissue at the present time.

## Concluding remarks

**Fig. 6.**
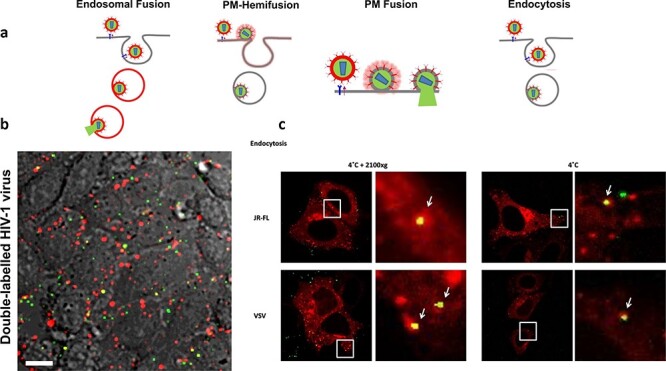
Real-time single virus tracking in live cells. (a) HIV-1 pseudoviruses can be labelled with lipophilic dyes such as DiD in the envelope and with Gag-eGFP that is cleaved in mature viruses. When hemifusion occurs in the plasma membrane, DiD signal dilutes and vanishes and the tracked HIV-1 particle changes colour from yellow to green. If full fusion occurs, the cleaved eGFP monomers are released to the cytosol and the green signal disappears. If the HIV-1 particle fuses within endosomal compartments, the DiD signal remains in the endosomal membrane upon hemifusion. Full fusion is detected when the eGFP signal disappears, and therefore the particle changes from yellow to red. (b) Example of TZM-bl cells exposed to double-labelled HIV-1 viruses. Scale bar = 10 μm. (c) Examples of double-labelled HIV-1 particles either exposed to viruses at 4°C and subject to spinoculation (2100 G, left panels) where endocytosis occurs for both HIV-1 particles pseudotyped with JRFL Env and VSG-G Env and only exposed to double-labelled HIV-1 virions exposed at 4°C (right panels). TZM-bl cells were expressing Rab5-mCherry to highlight early endosomal compartments. The micrographs were 40 × 40 μm.

Investigations aimed at identifying key mechanisms involved in HIV-1 penetration of vulnerable mucosal surfaces are critical in order to faithfully design novel therapeutic strategies that can efficiently prevent or treat acute HIV-1 infection. Unfortunately, despite the high volume of literature investigating the mucosal entry of HIV-1, the real-time, direct infection of HIV-1 in target cells at mucosal surfaces has not been observed. We believe that this is due to a previously heavy reliance of the current literature on animal and 2D culture models (Fig. 6), as well as their fixed preparations. Nevertheless, this information is vital to more effectively detail the precise biochemical, tissue-specific and host-specific factors that could alter target cell susceptibility and the course of HIV-1 infection of its initial susceptible population of target cells.

HIV-1, human immunodeficiency virus type-1.

It is true that much knowledge regarding the mucosal transmission of HIV-1 in the FGT and CRT has been acquired utilizing animal-based and 2D culture–based methods. Nevertheless, as mentioned previously, the utilization of organoid-based models can help close the gaps that remain from studies relying on these systems to study viral entry at mucosal surfaces. In particular, organoids may indeed create a platform enabling substantially greater complex modelling of HIV-1 transmission and infection by allowing the incorporation of commensal organisms, controlled inflammatory and biochemical contexts as well as target cell densities to be manipulated. As these methods become more established, they can be further applied to mucosal surfaces apart from the FGT and CRT, such as the penile foreskin and oral mucosa, where many studies remain to be conducted to fully elucidate the precise mechanisms of HIV-1 transmission. For example, although it is known that Langerhans cells, DCs and CD4^+^ T cells are found in the penile foreskin at more superficial surfaces and at higher densities in uncircumcised men, it is only speculated that these are the contributing factors to increased HIV-1 infection in these patients [131]. Additionally, although inner foreskin has been shown to be slightly more susceptible to HIV-1 infection, these results have not been corroborated by other studies without conflicting evidence [132]. These postulations require direct, observational evidence that can be acquired in organoid-based methods utilizing 3D imaging.

Advanced light microscopy techniques have been rapidly evolving to provide investigators with powerful tools to image cells in complex tissue environments with drastically advanced spatial and temporal resolutions and elucidate mechanistic aspects related to particular biological and pathological contexts in viral infection. In particular, we believe that label-free two-photon confocal microscopy and intravital Forster resonance energy transfer (FRET)-FLIM, on-line single-particle tracking and light sheet microscopy are at the forefront of techniques that could provide key information regarding HIV-1 entry at its mucosal surfaces. Thus, we hope that this review serves the scientific community not only to recognize the complexity of HIV-1 entry at prominent HIV-1 mucosal sites or why organoids should be utilized to fill lapses in knowledge of HIV-1 mucosal penetration but also to serve as a call for studies and collaborations multiplexing this microscopy technology with advanced organoid development of the FGT and CRT to uncover potential therapeutic targets which prevent the establishment of HIV-1 infection.

## Funding

This work has been supported by the National Institutes of Health Oxford-Cambridge Fellowship to C.A.C. S.P.-P. acknowledges funding from the Nuffield Department of Medicine leadership fellowship and all authors from the Wellcome Trust Core Award (203141). This work has also been supported by the European Research Council (ERC-2019-CoG-863869 FUSION to S.P.-P.).
